# The Story of the Insane from Year to Year

**Published:** 1901-02-02

**Authors:** 


### THE STORY OF THE INSANE FROM YEAR TO YEAR.

(Thirteenth Series.)

### THE SURREY COUNTY ASYLUM AT BROOKWOOD.

On January 1st, 1899, this asylum contained 1,088 patients, and by the end of the year the number had been reduced to 999. There were 215 first admissions, 45 re-admissions, 114 recoveries, 59 discharged relieved, and 100 deaths. The recoveries stand at 45·16 per cent. of the admissions, and the deaths at 9·25 per cent. on the average resident. The recoveries are considerably above the average, and the deaths slightly under it. Of the year's deaths 42 were caused by cerebral and spinal diseases, 6 by pneumonia, 18 by consumption, 2 by enteritis, and 1 by enteric fever. Of the year's admissions 13 owed their insanity to domestic trouble, 47 to mental anxiety, 9 to religion, and 8 to love affairs; intemperance in drink accounts for 43, and hereditary influence for 72, while influenza is set down as the cause in 10 cases. Unquestionably the latter has played an important part in the causation of insanity during the last ten years, although not perhaps to the extent which our statistics would lead us to believe, as it would seem tolerably certain that many of the cases would have given way under some other strain had the influenza been absent; but still it has been the sole cause in some. The reduction in numbers mentioned at the beginning of this notice was owing to the transference of 33 harmless chronic cases to the workhouses, and by the transference of 59 cases to the Gloucester Asylum, where their extra cost to the county will be something like £500 a year, a fact more pleasant for the Gloucestershire ratepayers to contemplate than for the Surrey. Dr. Barton gives a paragraph about the deaths, but says nothing about those from enteritis and enteric fever. The weekly charge for maintenance has been 10s. 6d. and 11s. per head.

### THE CRICHTON ROYAL INSTITUTION, DUMFRIES.

This asylum contained 757 patients on January 1st, 1899, and 749 on the last day of the year. There were 133 first admissions, 32 re-admissions, 70 recoveries, and 54 deaths. Calculated on the admissions of the year the recoveries reached the rather high percentage of 42·4, and calculated on the average number resident the deaths stood at 7·2 per cent. Of the deaths, 19 were caused by cerebral and spinal diseases, 11 by phthisis, 3 by bronchitis, 1 by pneumonia, and 1 by enteritis. The table showing the causes of insanity in those admitted is not given; but we learn from another part of the report that alcohol was the principal cause in one-fifth of the cases admitted. Very few asylums can report a diminution of the rate-supported patients; but this asylum can boast of being in that position; and Dr. Rutherford says "It would be gratifying to believe that the fewer admissions of rate-supported patients was due to the improved social conditions which undoubtedly exist as compared with twenty years ago." We wish we could think so; but when we hear that the average number of pauper admissions has gone down from 73 to 66, and then to 56, we feel sure there must be some other causes at work than improved sanitary conditions, better education, and higher wages; and Dr. Rutherford tells us that the reduced rate charged for the board of private patients has something to do with the fall in the admission rate of paupers; and when we realise that anyone who can pay £10 a year for his maintainance may avoid the stigma of pauperism, we can readily believe that we have here a potent factor in the reduction of rate-supported patients. It is most regrettable that a rich country like England possesses no institutions comparable to these Royal Asylums of Scotland. Undoubtedly much charitable work is done by the Crichton Institution; and we should much like to see in the next report a table giving the various rates of board, and the numbers of patients received at these rates.

### THE EASTERN COUNTIES ASYLUM FOR IDIOTS.

At this asylum, which is situated in Colchester, there were 250 patients at the beginning of 1899, and 254 at the end of the year. There were 33 admissions, 12 discharges, and 17 deaths. It is curious to notice that in asylums of this class the males greatly outnumber the females, whereas in county asylums the reverse is nearly always the case. The ages of those admitted varied, among the males, from 8 to 27 years, and among the females from 5 to 29. Mr. Turner tells us that in 10 of the year's admissions there have been definite signs of improvement, and that in 7 others improvement is distinctly noticeable. This shows what care, education, discipline, attention, and employment will do for the most unpromising class of human beings; but those not familiar with idiot asylums cannot understand the difficulties which have to be encountered by the staff in effecting such improvement. Much has been done lately to bring this asylum up to a high standard, and it now compares favourably with kindred institutions. From Mr. Owsley's report we learn that the death-rate was 6·7 per cent. of the average number resident. As is usual in idiot asylums consumption was the chief factor in the mortality; ten out of the seventeen deaths being due to it. The total income amounted to £8,997, and the expenditure to £8,396, thus permitting a sum of £600 to be placed to the reserve fund. The income is made up of £2,091 from annual subscriptions, £20 from donations, £995 from dividends, and £3,353 from payment cases. Regarding the private patients' payments we believe the board of management pursues a more enlightened policy than is found in some similar establishments; and manifestly the lower these payments can be made, consistently with outlay, the better will it be for the charity generally, because fewer cases will be thrown on it. The average weekly cost is a fraction under 12s., but this does not include such sums as must from time to time be expended in keeping the building in repair. Mr. Peckover has again shown his interest in the institution, and has undertaken to defray the cost of a connecting corridor between the schools and the main building.

### THE DERBY BOROUGH ASYLUM.

Here the numbers fell from 324 to 300 during the year 1899; but this reduction was owing to the transference of 42 patients to the Stafford Asylum. There were 82 first admissions; 16 re-admissions; 32 recoveries; 6 discharged relieved; and 38 deaths. The recoveries stand at 37·2 per cent. of the admissions, and the deaths at 11·2 per cent. on the average number resident. Of the deaths 23 were caused by cerebral and spinal diseases and only 1 by phthisis. Of the year's admissions 30 owed their insanity to moral causes; 20 to intemperance in drink, and 33 to hereditary influence. Dr. Macphail laments the hopeless nature of so many of the admissions, and certainly they were not a favourable lot to work on. Eleven were paralytics, 6 were epileptics, 4 were congenital imbeciles, 5 were over seventy years of age, 11 had been insane for years, and 4 were dying of malignant diseases. Here is a rich field for discovery and for pathological work likely to influence the recovery rate in some remote future. Altogether 11 per cent. of the patients admitted during 1899 died within that year. The low death rate from phthisis is, however, most satisfactory. We are glad to note that the medical officers continue their lectures to the staff, and that six additional attendants and nurses have obtained the certificate of the Medico-Psychological Association. We should be still more pleased to learn that a three-years' system of training was in force. The Committee record their "entire satisfaction with the manner in which Dr. Macphail continues to discharge the responsible and onerous duties which devolve on him." The weekly charge for maintenance is 10s. 6d. per head.

